# Gallic Acid Ameliorates the Inflammatory State of Periodontal Ligament Stem Cells and Promotes Pro-Osteodifferentiation Capabilities of Inflammatory Stem Cell-Derived Exosomes

**DOI:** 10.3390/life12091392

**Published:** 2022-09-06

**Authors:** Zhenning Dai, Ziyue Li, Weihan Zheng, Zi Yan, Lijun Zhang, Jiaxin Yang, Jing Xiao, Hanxiao Sun, Shiyu Li, Wenhua Huang

**Affiliations:** 1 Department of Stomatology, Guangdong Key Laboratory of Traditional Chinese Medicine Research and Development, The Fifth Clinical College of Guangzhou University of Chinese Medicine, Guangdong Second Traditional Chinese Medicine Hospital, Guangzhou 510095, China; 2 Guangdong Medical Innovation Platform for Translation of 3D Printing Application, The Third Affiliated Hospital of Southern Medical University, Southern Medical University, Guangzhou 510630, China; 3 Department of Anatomy, Guangdong Provincial Key Laboratory of Digital Medicine and Biomechanics, School of Basic Medical Sciences, Southern Medical University, Guangzhou 510515, China; 4 Institute of Genomic Medicine, College of Pharmacy, Jinan University, Guangzhou 510632, China; 5 Division of Spine Surgery, Section II, Department of Orthopedics, Guangdong Provincial Key Laboratory of Bone and Joint Degeneration Diseases, The Third Affiliated Hospital of Southern Medical University, Southern Medical University, Guangzhou 510630, China

**Keywords:** periodontitis, periodontal membrane stem cells, exosomes, gallic acid, osteodifferentiation, aerobic glucose metabolism, oxidative stress

## Abstract

The slow proliferation rate and poor osteodifferentiation ability of inflammatory periodontal membrane stem cells extracted from periodontitis tissues (i-PDLSCs) account for poor efficiency in treating inflammatory bone loss. Exosomes reportedly have inducible and relatively stable components, allowing them to promote inflammatory bone repair, but obtaining i-PDLSCs exosomes with the ability to promote osteodifferentiation is challenging. In the present study, i-PDLSCs were extracted from periodontal membrane tissues of patients with severe periodontitis, and in vitro induction with gallic acid (GA) significantly promoted the proliferative activity of i-PDLSCs at a concentration of 10 mM, with TC_0_ of 11.057 mM and TC_50_ of 67.56 mM for i-PDLSCs. After mRNA sequencing, we found that GA could alleviate oxidative stress in i-PDLSCs and increase its mitochondrial membrane potential and glucose aerobic metabolism level, thus promoting the osteodifferentiation of i-PDLSCs. After exosomes of i-PDLSCs after GA induction (i-EXO-GA) were isolated by differential centrifugation, we found that 200 ug/mL of i-EXO-GA could remarkably promote the osteodifferentiation of i-PDLSCs. Overall, our results suggest that GA induction can enhance the proliferation and osteodifferentiation in primary cultures of i-PDLSCs in vitro, mediated by alleviating oxidative stress and glycometabolism levels in cells, which further influences the osteodifferentiation-promoting ability of i-EXO-GA. Overall, we provide a viable cell and exosome induction culture method for treating inflammatory alveolar defects associated with periodontitis.

## 1. Introduction

Periodontitis is a chronic inflammatory disease associated with bacterial biofilm and characterized by the destruction of the tooth-supporting tissues, which can eventually lead to loose teeth or even edentulism [1]. In the pathogenesis of periodontitis, multiple inflammatory factors are activated [2], resulting in pathological resorption of alveolar bone, known as alveolar inflammatory bone loss [3]. Furthermore, bone loss results in insufficient bone volume in the alveolar bone, significantly limiting the feasibility of dental implantation. It has been established that periodontitis can affect the efficacy of guided bone regeneration (GBR) treatment [4].

Over the years, periodontal ligament stem cells (PDLSCs) have been widely applied as seed cells for tissue-engineered repair of bone deficiencies and alveolar bone defects in studies on alveolar bone tissue engineering and regeneration [5]. Contemporarily, it has been shown to promote periodontal tissue regeneration with good osteodifferentiation potential. However, the extraction of therapeutically available PDLSCs in periodontitis is unpractical since the extracted cells mostly exhibit an inflammatory phenotype with poor proliferative activity and osteodifferentiation potential [6]. Another paradox is that PDLSCs transferred into the periodontitis environment can be regulated by inflammatory factors and eventually fail to promote alveolar bone healing. In recent years, it has been proposed that exosomes (EXO) secreted by PDLSCs are bioactive components in the construction of cell-free tissue engineering scaffolds [7]. Exosomes of PDLSCs are extracellular vesicles with a diameter of 30–150 nm, rich in proteins, miRNA, RNA, lipids, and DNA. Current evidence suggests that transplanted PDLSCs promote tissue regeneration mainly through paracrine action. Exosomes play a key role in stem cell therapy as a major component of the stem cell secretion system [8,9]. Therefore, PDLSCs exosomes can replace PDLSCs during therapy and compensate for their limitations, such as long primary culture time and intolerance to long-term storage. Interestingly, exosomes exhibit a relatively stable composition after being secreted and are not subject to changes in the inflammatory environment [10]. Therefore, exosomes from PDLSCs have huge prospects for treating inflammatory bone loss associated with periodontitis. Nevertheless, it remains unclear how efficient exosomes of PDLSCs can be obtained from periodontitis tissues.

We hypothesized that the culture of inflammatory PDLSCs with improved inflammatory phenotype and osteodifferentiation could facilitate the acquisition of exosomes that promote osteodifferentiation. It has been established that the Chinese medicine, Xipayi mouth rinse, made from gallic, is effective against oral bacteria that cause simple gingivitis and mild periodontitis [11]. Moreover, it can prevent and treat oral bacterial infectious diseases and reduce plaque and gingival bleeding. Gallic acid (GA) is a polyphenolic organic compound reported to have various biological functions such as anti-inflammatory, anti-tumor, and antioxidant damage [12]. GA can alleviate periodontitis by inhibiting nuclear factor (NF-κB) upregulation and phosphorylation induced by lipopolysaccharide (LPS). It inhibits the excessive release of inflammatory cytokines IL-1β and TNF-α and reduces bone destruction, while GC may reduce LPS-induced osteoclast activation and alveolar bone resorption by upregulating the OPG/s-RANKL pathway to achieve therapeutic effects [13,14]. However, the effect of GCon cellular function and exosomes of inflammatory PDLSCs is unknown. In our study, we implemented an array of experiments to assess the osteodifferentiation promoting ability of GA-induced i-PDLSCs exosomes (i-EXO-GA) in vitro, which may provide a new strategy for the treatment of inflammatory bone loss caused by periodontitis.

## 2. Materials and Methods

### 2.1. Materials

Gallic acid (Yuanye BioTech, Shanghai, China); Type I collagenase, CAT ELISA kit, SIRT1 ELISA kit (Sigma, St. Louis, MO, USA); Exosome-free serum (SBI, Palo Alto, CA, USA); Medium DMEM, DPBS solution (Gibco, Grand Island, NY, USA); chloroform, isopropanol (MackLin, Shanghai, China); SOD1, SOD2 ELISA kit (Ray Biotech, Guangzhou, Guangdong, China); SIRT3 ELISA kit (Abnova, Taipei, Taiwan, China); MTT, ATP kit, alizarin red dye solution (Solarbio, Beijing, China); lactic acid content kit (Ruixin Biotech, Quanzhou, Fujian, China); JC-1 kit (Beyotime, Shanghai, China); carbonyl cyanide m-chlorophenylhydrazone CCCP (BestBio, Shanghai, China); MDA kit (Dojindo, Kumamoto, Japan); SOD kit (Jingmei Biological, Yancheng, Jiangsu, China); BCA protein concentration kit (Thermo, Waltham, MA, USA); alkaline phosphatase staining kit (Beyotime, Shanghai, China); anti-CD81, Anti-CD9, Anti-ALIX, Anti-β-actin (Abcam, Cambridge, MA, USA). Primers were ordered from Shanghai Sangon Biotech.

### 2.2. Experimental Methods

#### 2.2.1. Primary Cell Extraction and Culture of PDLSCs

The present study was approved by the Ethics Committee of Guangdong Second Hospital of Traditional Chinese Medicine. Primary cell extraction was conducted after obtaining informed consent from the patients. Healthy periodontal ligament stem cells (h-PDLSCs) were harvested from the periodontal ligament tissues of healthy teeth extracted before orthodontic treatment of 3 patients. Inflammatory periodontal ligament stem cells (i-PDLSCs) were harvested from the inflammatory periodontal ligament tissues of 3 patients with stage III periodontitis (alveolar bone loss extending to mid-third of root and beyond, tooth loss due to periodontitis of ≤4 teeth, probing depth ≥6 mm, and vertical bone loss ≥3 mm). PDLSCs were not harvested from patients with diabetes, metabolic diseases, recent hormone use, tumors, etc. During primary cell extraction and cell culture, 1/3 of the periodontal ligament tissue in the root was scraped and washed with DPBS solution containing BI 1-antibody and 3% gentamicin for 1 min. The periodontal ligament tissue was added into DPBS containing 3 mg/mL Collagenase I and digested in a water bath at 37 °C for 1 h. After digestion, the cells were filtered through a 70 μm filter membrane to obtain the cell suspension. After centrifugation at 100× *g* for 5 min, the cells were resuspended and precipitated with a complete medium (DMEM medium containing 10% exosome-free serum and 1% penicillin-streptomycin). The cell density was adjusted to 1 × 10^4^ cells/mL. The cells were inoculated in a T25 cell culture flask and placed in a 5% CO_2_ incubator at 37 °C. After 24 h, the non-adherent cells were removed by completely changing the liquid, and the liquid was completely changed once in the following 2–3 d. The cells were subcultured when the cell density reached 80%.

#### 2.2.2. GA Induction Culture

The second generation of i-PDLSCs was seeded in 6-well plates, and the complete medium containing different concentrations of GA was added for culture induction, which was named i-PDLSCs-GA.

#### 2.2.3. MTT Cytotoxicity Assay

The second generation of i-PDLSCs was inoculated in 96-well plates and induced with different concentrations of GA for 24 h. h-PDLSCs incubated in a complete culture medium were used as the control group. The MTT assay was used to detect the cytotoxicity of different concentrations of GA on i-PDLSCs. GA was dissolved in DPBS solution and diluted to 400, 200, 100, 50, 25, 10, 5, 2.5, 1.25, and 1 mM in a complete culture medium to induce i-PDLSCs. According to the kit instructions, 20 μL MTT working solution was added to each well. After 4 h of culture and centrifugation at 2000× *g* for 10 min, 200 μL DMSO was added to each well and fully oscillated. The absorbance value was read at 490 nm using a microplate reader. Cell survival rate (%) = (i-PDLSCs-GA group average OD value/h-PDLSCs group average OD value) × 100%. The maximum toxic concentration (TC0) and half toxic concentration (TC50) were calculated by the probit regression function of SPSS (version 24.0) software (IBM, Armonk, NY, USA).

#### 2.2.4. mRNA Sequencing

RNA was extracted from h-PDLSCs, i-PDLSCs, and i-PDLSCs-GA cultured for 3 d, and mRNA sequencing was performed. Total RNA was extracted by the TRIZOL method. Cells were passaged into a T75 cell culture flask, and 5 mL TRIZOL was added. An amount of 4 mL was frozen in liquid nitrogen for mRNA sequencing. A total of 250 μL chloroform was added to 1 mL solution and shaken until a pink color was observed, kept at room temperature for 10 min, and centrifuged at 12,000× *g* for 5 min at 4 °C. The supernatant was taken into the EP tube and added to 500 μL isopropanol; the mixture was shaken and allowed to stand at room temperature for 15 min. An amount of 1 mL of 75% ethanol was added, and the precipitate was washed and centrifuged at 4 °C for 10 min at 12,000× *g*. The supernatant was removed, and 50 μL DEPC water was added to dissolve RNA after being dried in the ventilation cabinet. An amount of 1 μL was taken for Nanodrop detection. An OD value of 1.8–2.0 was acceptable, and the remaining samples were stored at −80 °C for further use. The purity and integrity of total RNA were analyzed by Agilent 2100 BioAnalyzer system, and samples with RNA integrity number ≥8.0 were selected for further detection. A lysing reagent was added to lyse the mRNA into short fragments at moderate temperature in a thermostatic mixer. A one-stranded cDNA was synthesized using the interrupted mRNA as a template, and a two-stranded synthesis reaction system was formulated to synthesize two-stranded cDNA. After purification and recovery, we conducted sticky end repair and addition of base “A” to the 3’ end of cDNA with ligated connectors, followed by fragment size selection and PCR amplification for library construction according to the kit instructions. The constructed libraries were quality-checked by Agilent 2100 Bioanalyzer and Qubit and sequenced using Illumina sequencers. The data obtained from Illumina HiSeqTM 2000 sequencing, called Raw reads or Raw data, were then subjected to quality control (QC) to determine if the sequencing data is suitable for subsequent analysis. After the above QC, a series of follow-up analyses such as gene and transcript quantification, various analyses based on gene expression levels (principal component, correlation, condition-specific expression, differential gene screening, etc.), exon quantification, gene structure optimization, variable splicing, new transcript prediction and annotation, SNP detection, Indel analysis, gene fusion, etc. were performed. The differentially expressed genes (DEGs) among the screened samples were analyzed by GO functional significance enrichment analysis, pathway significance enrichment analysis, clustering, protein interaction network, and transcriptional factors for more in-depth mining.

#### 2.2.5. Quantification of DEG-Related Proteins by ELISA

DEGs were screened according to the mRNA sequencing results, and the protein expression levels corresponding to the DEGs were verified by ELISA. The h-PDLSCs, i-PDLSCs, and i-PDLSCs-GA were cultured for 3 d, and SOD1, SOD2, CAT, SIRT1, and SIRT3 levels were determined according to the kit’s instructions.

#### 2.2.6. Detection of Mitochondrial Membrane Potential

The JC-1 mitochondrial membrane potential kit was used to detect h-PDLSCs, i-PDLSCs, and i-PDLSCs-GA cultured for 3 days. The cells in each group were seeded in a confocal 6-well plate. JC-1 staining was conducted according to the kit’s instructions and added to the plate. The cells were incubated at 37 °C for 15 min. The negative control group was set as h-PDLSCs-CCCP with 10 μM CCCP. An inverted laser confocal microscope was used to stimulate and photograph at 488 and 594 nm. It has been established that JC-1 exists in the mitochondrial matrix as a polymer when the mitochondrial membrane potential is high, and the polymer fluorescence can be measured at 594 nm. However, JC-1 exists as a monomer when the mitochondrial membrane potential is low and cannot be aggregated in the mitochondrial matrix. JC-1 is mostly dissociated in the cytoplasm, and the monomer fluorescence can be measured at 488 nm. Fluorescence images were quantified by the ratio of two fluorescence channels (594/488 nm).

#### 2.2.7. Detection of Oxidative Stress Level

MDA and SOD levels were detected in h-PDLSCs, i-PDLSCs, and i-PDLSCs-GA cultured for 3 days using ELISA kits.

#### 2.2.8. Detection of Glycometabolism Level

The metabolites of h-PDLSCs, i-PDLSCs, and i-PDLSCs-GA cultured for 3 d were determined by ATP and lactic acid detection kits. The activity of the glycolysis rate-limiting enzyme was determined by 6-phosphate fructose kinase (PFK-1) activity and pyruvate kinase (PK) activity detection kit. The expression levels of citrate synthase (CS) and malate dehydrogenase (IDH), NADH dehydrogenase 1-β3 (NDUFB3), and succinate dehydrogenase complex β subunit (SDHB) were detected by RT-qPCR. The reaction system included SsoFast Eva Green Supermix 5 μL, upstream primer (10 μM) 0.5 μL, downstream primer (10 μM) 0.5 μL, cDNA template 2 μL, and ultrapure water 2 μL. The reference gene β-tubulin was used for data normalization. The expression of related genes was calculated by the 2^−ΔΔCT^ method. The primer sequences are listed in Table 1.

#### 2.2.9. Detection of Osteodifferentiation Level

Alkaline phosphatase (ALP) staining was performed on h-PDLSCs, i-PDLSCs, i-PDLSCs-GA, and h-PDLSCs-CCCP with 10 μM CCCP cultured for 7 d. The cells in each group were inoculated on a 12-well plate for 7 d. The plate cells were washed with PBS and fixed with 4% paraformaldehyde for 15 min. The color buffer, BCIP solution, and NBT solution were prepared into a working solution according to 300:1:2. An amount of 500 μL working solution was added to each well and incubated at room temperature for 30 min. The cells were washed with distilled water and photographed with a micro camera.

The h-PDLSCs, i-PDLSCs, i-PDLSCs-GA, and h-PDLSCs-CCCP cultured for 14 d were stained with alizarin red. The cells in each group were inoculated in 12-well plates for 14 d. The cells in the well plates were washed with PBS, fixed with 4% paraformaldehyde for 15 min, and added with 1 mL alizarin red staining solution. After static staining at room temperature for 30 min, the cells were washed with distilled water and photographed with a micro-camera.

#### 2.2.10. Extraction of Exosomes by Differential Centrifugation

h-PDLSCs, i-PDLSCs, and i-PDLSCs-GA cells cultured for 3 d were inoculated into 10 T75 culture flasks. After the cells were cultured to 80% confluence, they were washed twice with PBS. The complete medium containing 10% exosome-free serum and 1% double antibody was used for total exchange. The medium in each flask was 50 mL. After 72 h of culture, the supernatant of the cell culture medium (500 mL in each group) was collected for exosome extraction. The supernatant was centrifuged at 300× *g* for 10 min at 4 °C to transfer the supernatant and then centrifuged at 10,000× *g* for 10 min at 4 °C to remove dead cells and cell debris. The supernatant was carefully collected and transferred to a 50 mL open-ended ultracentrifugal tube for 4 °C and 100,000× *g* for 70 min, and the supernatant was discarded. The precipitate was suspended with 2 mL DPBS and then transferred to a 12.5 mL tube for centrifugation at 4 °C for 70 min at 100,000× *g*. Each centrifuge tube was suspended with 200 μL PBS. After filtering with a 0.22 μm pore size, it was loaded with an EP tube and stored at −80 °C to avoid repeated freezing and thawing. Exosomes extracted from the supernatants of h-PDLSCs, i-PDLSCs, and i-PDLSCs-GA cell culture medium were recorded as h-EXO, i-EXO, and i-EXO-GA, respectively.

#### 2.2.11. Exosomes Identification

(1) Determination of BCA protein concentration: An amount of 20 μL exosome solution from each group was taken for reaction with the BCA working solution and incubated at 37 °C for 30 min. The OD values of the sample group and the standard group at 562 nm were read by the microplate reader, the standard curve was plotted, and the protein concentration of the sample was calculated. The samples were adjusted to the same protein concentration for subsequent detection. (2) Identification of exosome markers by Western blot: A total of 100 μL exosome extracts from each group were mixed with 20 μL protein sample buffer and heated at 95 °C for 5 min. The protein separated by SDS polyacrylamide gel electrophoresis was transferred to PVDF membranes incubated in 5% skim milk powder (prepared with PBS solution) at room temperature for 1 h. They were then incubated with a 1:1000 diluted primary antibody (Anti-CD81, Anti-CD9, Anti-ALIX, Anti-β-actin) overnight at 4 °C, followed by a 1:5000 dilution horseradish peroxidase-labeled secondary antibody at room temperature for 1 h. ECL luminescent solution was added and incubated for 3 min for chemiluminescence imaging. (3) Particle size analysis: An amount of 1 mL exosome samples was slowly injected into the Nanosight nanoparticle size analyzer, and the thermometer probe was placed in the copper hole. After adjusting to the appropriate focal length, the particle size and concentration were determined.

#### 2.2.12. Coculturing of i-PDLSCs and Exosomes

h-EXO and i-EXO exosomes were adjusted to 300 μg/mL in a complete culture medium, and i-EXO-GA exosomes were adjusted to gradient concentrations of 100, 200, and 300 μg/mL. After digestion, i-PDLSCs were resuspended in an exosome-containing medium and inoculated in 12-well plates.

#### 2.2.13. Detection of Osteodifferentiation Index

Alkaline phosphatase (ALP) staining: ALP staining was performed on the cocultured cells of each group at 7 d, using the same method as in Section 2.2.9; (2) Alizarin red staining: The cells cocultured in each group were stained with alizarin red on the pore plate at 14 d; (3) RT-PCR: RT-qPCR was used to detect the expression levels of Runt-related transcription factor 2 (RUNX2), osteocalcin (OCN), and type I collagen (COL-I) in the cocultured cells of each group at 0, 7, 14, and 21 d. The 12-well plate cells were transferred to the EP tube, frozen and crushed into powder in liquid nitrogen, and the total RNA was extracted by RNA kit. The Prime ScriptTM RT Master Mix reverse transcription kit was used for reverse transcription, and the primers were added to the real-time fluorescence quantitative PCR analyzer. The measured data were used to calculate the expression of related genes by the 2^−ΔΔCT^ method. The primer sequences used are listed in Table 2.

#### 2.2.14. Statistical Analysis

Graphpad prism 9.3 software was used for statistical analysis. The data were expressed as x ± s. One-way analysis of variance was used to compare multiple groups of samples. A *p*-value < 0.05 was statistically significant.

## 3. Results

### 3.1. GA Promotes the Proliferation Activity of i-PDLSCs

**Figure 1:**
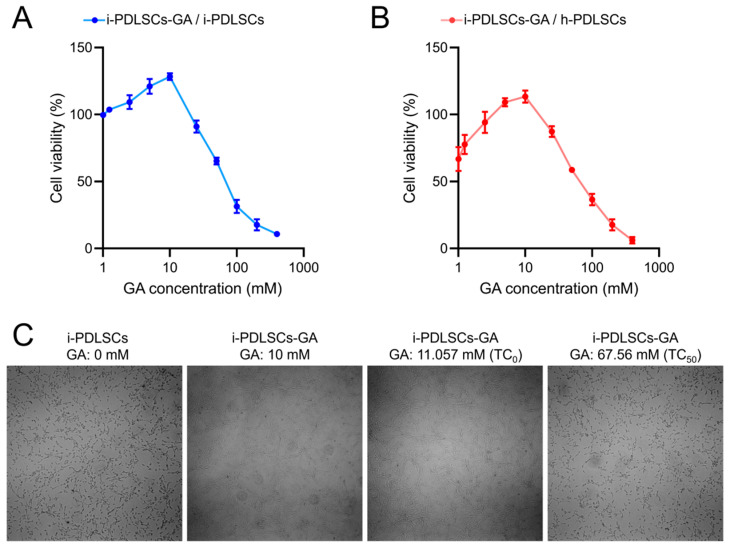
The effect of GA on the of PDLSCs proliferation detected by MTT. (**A**) The OD value ratio of i-PDLSCs-GA to i-PDLSCs; (**B**) The OD value ratio of i-PDLSCs-GA to h-PDLSCs; (**C**) The micrographs of i-PDLSCs-GA.

The MTT assay was conducted on i-PDLSCs induced by different concentrations of GA (Figure 1). GA induction promoted the proliferation of i-PDLSCs, especially with 10 mM GA. In contrast, 25 mM GA induced a relatively weak stimulatory effect on cell proliferation. We found that the maximum non-toxic concentration (TC0) and the half toxic concentration (TC50) of GA on i-PDLSCs were 11.057 and 67.56 mM, respectively (Figure 1A). As shown in Figure 1B, the proliferation activity of i-PDLSCs was lower than that of h-PDLSCs, and the cell survival rate was 66.87% of h-PDLSCs. When the GA concentration was 5 and 10 mM, i-PDLSCs-GA exhibited higher proliferation activity than h-PDLSCs. However, when the GA concentration was above 25 mM, i-PDLSCs-GA exhibited lower proliferation than h-PDLSCs, indicating that high concentrations of GA were cytotoxic. Accordingly, 10 mM GA was selected for culture induction in subsequent experiments. The micrographs of i-PDLSCs induced by different concentrations of GA (Figure 1C) showed that cell proliferation was not significantly different from the uninduced group when the GA concentration was 10 mM and 11.057 mM, and the cell proliferation was significantly inhibited when the GA concentration was 67.56 mM.

### 3.2. mRNA Sequencing Showed That the GA-Induced Culture Improved Oxidative Stress and Glycometabolism of i-PDLSCs

**Figure 2:**
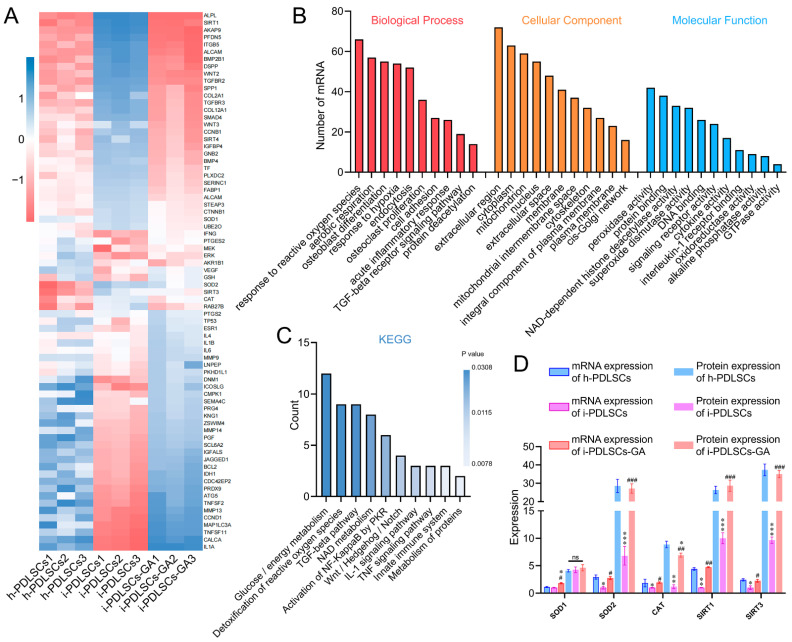
The effect of GA-induced culture by mRNA-Seq. (**A**) Heat map of differentially expressed genes; (**B**) GO enrichment analysis of differentially expressed genes; (**C**) KEGG enrichment analysis of DEGs; (**D**) ELISA detection of protein expression levels of differentially expressed genes, ns: *p* > 0.05, *, **, ***: compared with h-PDLSCs group *p* < 0.05, *p* < 0.01, *p* < 0.001, #, ##, ###: compared with i-PDLSCs group *p* < 0.05, *p* < 0.01, *p* < 0.001.

The mRNA sequencing results of h-PDLSCs, i-PDLSCs, and i-PDLSCs-GA on day 3 of GA induction are shown in Figure 2. A total of 1145 genes were detected by sequencing, and 74 DEGs exhibited a two-fold difference in expression between both groups (Figure 2A). BMP2B1, COL2A1, SOD2, SIRT3, CAT, and RAB27A exhibited high expression in h-PDLSCs. Moreover, IL1A, TNSF11, TNFSF2, MMP13, MEK, and DNM1 were highly expressed in i-PDLSCs. Finally, ALP, SIRT1, AKAP9, PFDN5, and ITGB5 were highly expressed in i-PDLSCs-GA. Gene ontology (GO) analysis (Figure 2B) of DEGs was conducted in terms of biological process, cellular component, and molecular function. The DEGs were significantly enriched in biological processes, including hypoxia, oxidative stress, aerobic respiration, osteoblast differentiation, and acute inflammation. Moreover, the DEGs were significantly enriched in cell components, including extracellular regions, cytoplasm, mitochondria, and nucleus. The molecular function of differentially expressed genes was mainly related to peroxidase activity, deacetylase activity, and superoxide dismutase activity. KEGG pathway enrichment analysis was used to describe the signaling pathways involved in differentially expressed genes (Figure 2C). The main signaling pathways involved include glucose/energy metabolism, anti-oxidative stress, TGF-β signaling pathway, NAD metabolism, etc. The expression levels of SOD1, SOD2, CAT, SIRT1, and SIRT3 in each group were verified by ELISA (Figure 2D). The results showed no significant difference in SOD1 protein expression among the three groups. The protein expression level in h-PDLSCs and i-PDLSCs-GA was significantly higher than in i-PDLSCs (*p* < 0.05). The sequencing results showed that high expression of inflammation-related genes in i-PDLSCs after GA treatment could cause changes in cellular oxidative stress, glycometabolism, osteodifferentiation, etc.

### 3.3. GA Treatment Alleviates Oxidative Stress in i-PDLSCs

**Figure 3:**
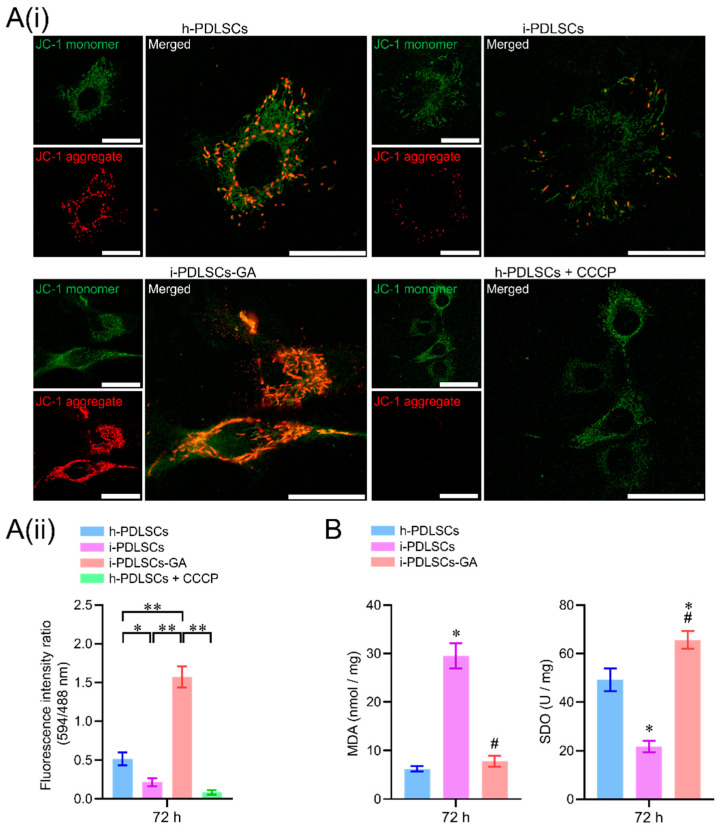
The effects of GA-induced culture on mitochondrial membrane potential and oxidative stress levels. (**A**(**i**)) fluorescence image of mitochondrial membrane potential JC-1 staining, with a scale of 10 μm; (**A**(**ii**)) fluorescence ratio quantitative analysis of mitochondrial membrane potential JC-1 staining, *: *p* < 0.05, **: *p* < 0.01; (**B**) ELISA detection of oxidative stress indexes MDA and SOD, *: *p* < 0.05 compared with h-PDLSCs group, #: *p* < 0.05 compared with i-PDLSCs group.

On day 3 of GA induction, the mitochondrial membrane potential, malondialdehyde (MDA), and superoxide dismutase (SOD) of h-PDLSCs, i-PDLSCs, and i-PDLSCs-GA were analyzed (Figure 3). As seen in Figure 3A(i), during mitochondrial membrane potential detection, the JC-1 probe could not enter the mitochondria at low mitochondrial membrane potential, showing green fluorescence JC-1 monomer. When the mitochondrial membrane potential was high, the JC-1 probe aggregated in the mitochondrial matrix in the form of polymers, showing red fluorescence. The results showed that the i-PDLSCs group and the h-PDLSCs + CCCP group treated with mitochondrial depolarization inducer CCCP had higher green fluorescence intensity and lower mitochondrial membrane potential. In comparison, the red fluorescence intensity of h-PDLSCs and i-PDLSCs-GA increased significantly. As shown in Figure 3A(ii), quantitative analysis of fluorescence ratio showed that the mitochondrial membrane potential of i-PDLSCs-GA cells induced by GA was significantly higher than i-PDLSCs and h-PDLSCs + CCCP (*p* < 0.05). During the quantification of oxidative stress indexes (Figure 3B), ELISA of oxidative stress marker MDA and anti-oxidative stress marker SOD showed that MDA and SOD of i-PDLSCs-GA were significantly different from i-PDLSCs (*p* < 0.05), and their SOD indexes were significantly higher than h-PDLSCs (*p* < 0.05). The above results suggested that the mitochondrial membrane potential of i-PDLSCs was low, and the oxidative stress level was high. After GA induction culture, the mitochondrial membrane potential and oxidative stress levels were significantly improved.

### 3.4. Enhancement of Glycometabolism in i-PDLSCs Induced by GA

**Figure 4:**
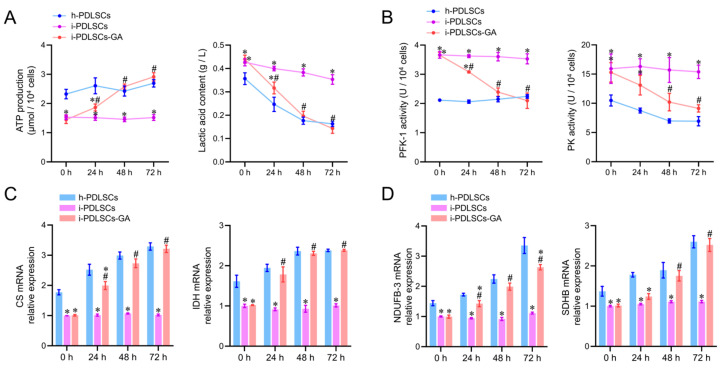
The effects of GA-induced culture on glycometabolism. (**A**) Determination of ATP production and lactic acid content; (**B**) ELISA detection of PFK-1 and PK enzyme activity; (**C**,**D**) RT-qPCR detection of CS, IDH, NDUFB-3, and SDHB. *: *p* < 0.05 compared with h-PDLSCs group, #: *p* < 0.05 compared with i-PDLSCs group.

On day 3 of culture induction with GA, the glycometabolism products, glycolytic enzyme activity, tricarboxylic acid cycle enzyme mRNA levels, and oxidized phosphorylase complex subunit mRNA levels in h-PDLSCs, i-PDLSCs, and i-PDLSCs-GA were analyzed (Figure 4). As shown in Figure 4A, the ATP production in i-PDLSCs was lower than in h-PDLSCs, and ATP production in i-PDLSCs-GA increased within 72 h, reaching a level similar to h-PDLSCs at 48 h. On the contrary, the lactic acid content in i-PDLSCs-GA decreased within 72 h. The activities of glycolytic rate-limiting enzymes 6-phosphofructose kinase (PFK-1) and pyruvate kinase (PK) are shown in Figure 4B. The enzyme activities in i-PDLSCs-GA decreased within 72 h, reaching a level similar to h-PDLSCs at 48 h. As shown in Figure 4C, the mRNA expression of citrate synthase (CS) and malic dehydrogenase (IDH), the key enzymes of the tricarboxylic acid cycle, were detected by RT-qPCR. The results showed that the expression of CS and IDH was upregulated in the GA-induced culture. Results in h-PDLSCs and i-PDLSCs-GA were significantly different from i-PDLSCs after 24 h (*p* < 0.05). As shown in Figure 4D, RT-qPCR was used to detect the mRNA expression of NADH dehydrogenase 1-β3 (NDUFB3) and succinate dehydrogenase complex β subunit (SDHB). NDUFB-3 expression in h-PDLSCs and i-PDLSCs-GA was significantly different than in i-PDLSCs after 24 h (*p* < 0.05). SDHB levels in i-PDLSCs-GA were significantly higher than in i-PDLSCs after 48 h (*p* < 0.05). The above results showed that the aerobic glucose metabolism in i-PDLSCs was significantly increased after GA induction for 24 h.

### 3.5. Effect of Oxidative Stress and Aerobic Glucose Metabolism on Osteodifferentiation of i-PDLSCs

**Figure 5:**
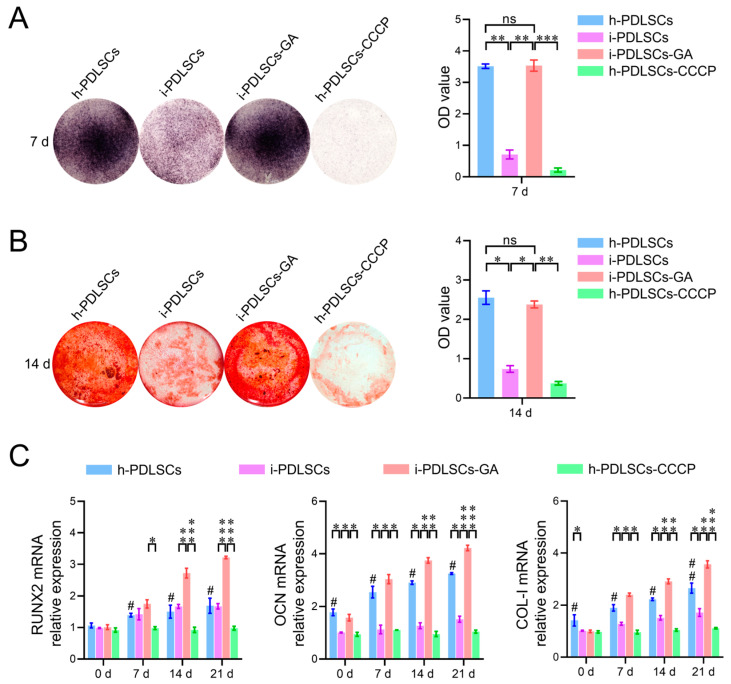
The effects of GA-induced culture on the level of osteodifferentiation. (**A**) Quantitative analysis of ALP staining and staining intensity; (**B**) Alizarin red staining and quantitative analysis of staining intensity. ns: *p* > 0.05, *: *p* < 0.05, **: *p* < 0.01, ***: *p* < 0.001; (**C**) RT-qPCR detection of osteodifferentiation-related genes on days 0, 7, 14, and 21. ns: *p* > 0.05, #: h-PDLSCs-CCCP group compared with h-PDLSCs group *p* < 0.05; ##: h-PDLSCs-CCCP group compared with h-PDLSCs group *p* < 0.01; *: *p* < 0.05, **: *p* < 0.01, ***: *p* < 0.001.

The results of cellular alkaline phosphatase (ALP) staining on day 7 of GA induction, alizarin red staining, and osteodifferentiation-related gene expression level assay on day 14 are shown in Figure 5. We found that h-PDLSCs and i-PDLSCs-GA exhibited higher ALP levels while i-PDLSCs had lower ALP levels (Figure 5A). Accordingly, almost no positive ALP staining was observed when h-PDLSCs-CCCP were treated with a sublethal dose (10 μM) of CPPP, a mitochondrial depolarization inducer. As shown in Figure 5B, alizarin red staining showed a similar trend, with many positively stained mineralized nodules in h-PDLSCs and i-PDLSCs-GA, few mineralized nodules in i-PDLSCs, and the lowest level of mineralized nodules in h-PDLSCs-CCCP. As seen in Figure 5C, there were no statistical differences in the expression of RUNX2 in the h-PDLSCs and i-PDLSCs groups on days 0, 7, 14, and 21, while the OCN and COL-I levels were lower in the i-PDLSCs group on days 14 and 21, and the h-PDLSCs- CCCP group had the lowest expression. On days 14 and 21, the expression of each osteodifferentiation-related gene was significantly higher in the i-PDLSCs-GA group, showing a more significant contribution to osteodifferentiation. It can be seen that alleviated mitochondria-related oxidative stress and enhanced aerobic glucose metabolism in the GA induction culture promoted the osteodifferentiation of i-PDLSCs-GA, while the loss of membrane potential of mitochondria after CCCP treatment significantly reduced the level of differentiation of h-PDLSCs, which exhibited good osteodifferentiation.

### 3.6. Identification of PDLSCs Exosomes

**Figure 6:**
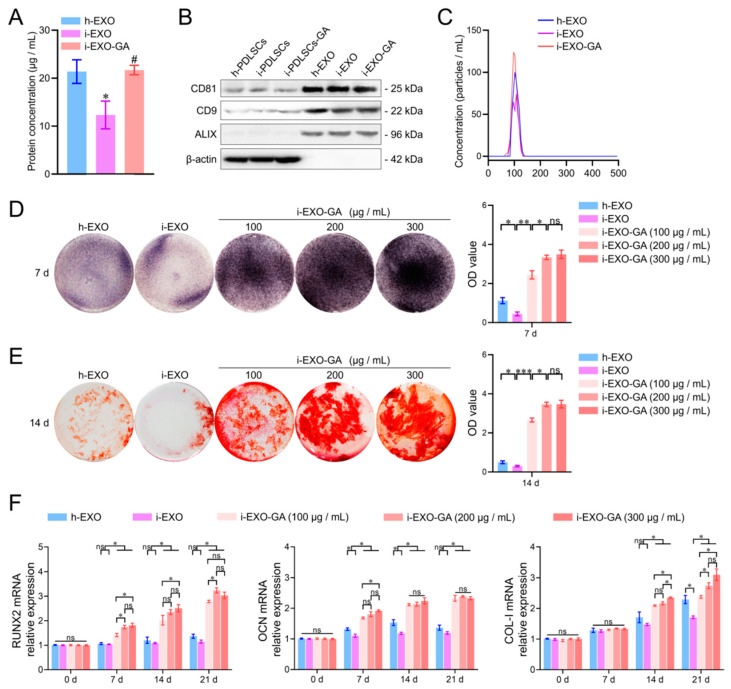
Exosome characterization, osteodifferentiation function detection and concentration screening. (**A**) BCA protein concentration detection of exosome samples, #: compared with the i-PDLSCs group, *p* < 0.05, *: compared with the h-PDLSCs group, *p* < 0.05; (**B**) Western blot detection of exosome markers; (**C**) NTA detection of exosome samples; (**D**) ALP staining and quantitative analysis of staining intensity of i-PDLSCs co-cultured with exosomes on day 7. (**E**) Alizarin red staining and quantitative analysis of staining intensity of i-PDLSCs co-cultured with exosomes on day 14. (**F**) RT-qPCR detection of osteodifferentiation-related genes cocultured with i-PDLSCs and exosomes on days 0, 7, 14 and 21. ns: *p* > 0.05, *: *p* < 0.05, **: *p* < 0.01, ***: *p* < 0.001.

Exosomes were extracted from the culture supernatants of h-PDLSCs, i-PDLSCs, and i-PDLSCs-GA cells by differential centrifugation and named h-EXO, i-EXO, and i-EXO-GA, respectively (Figure 6). The BCA protein assay was used to detect the content of exosomes in the supernatant of cell culture medium per unit volume. The results showed no significant difference between h-EXO and i-EXO-GA (Figure 6A) exosomes isolated from the supernatant of the cell culture medium, while levels in i-EXO were significantly less than the two groups (21.38 ± 2.01, 21.69 ± 0.80 and 12.33 ± 2.37 μg/mL, respectively). The surface markers CD81, CD9, and ALIX were detected, and β-actin was not detected in exosome samples by Western blot (Figure 6B, supplementary materials). The NTA particle size analysis results of exosomes are shown in Figure 6C. It can be seen that the particle sizes of the three groups of exosomes ranged from 30 to 150 nm, consistent with the particle size range of exosomes. However, i-EXO contained fewer particles than the other two groups. The above results substantiated that exosomes were successfully isolated from the supernatant of the cell culture medium.

### 3.7. The Function of i-EXO-GA in Promoting Osteodifferentiation and Concentration Screening

The concentrations of h-EXO and i-EXO were adjusted to 300 μg/mL, and the concentration gradients of i-EXO-GA were set to 100, 200, and 300 μg/mL. Coculture with i-PDLSCs was carried out in vitro, and the osteodifferentiation-related genes were quantified (Figure 6). The ALP staining results on day 7 are shown in Figure 6D. A high concentration of i-EXO induced a disproportionate decrease in ALP staining of i-PDLSCs cells. The high concentration of h-EXO could induce ALP expression, but the staining intensity was lower than in the i-EXO-GA group. i-EXO-GA concentration was positively correlated with ALP staining intensity. Alizarin red staining results on day 14 were consistent with ALP staining, as shown in Figure 6E. The quantification of osteodifferentiation-related gene expression at days 0, 7, 14, and 21 are shown in Figure 6F. We found that the expression of RUNX2 at each time point was comparable between h-EXO and i-EXO groups. On days 7 and 14 of OCN, and day 21 of the COL-I group, h-EXO showed a more obvious osteodifferentiation-promoting ability than i-EXO, indicating that h-EXO yielded a definite osteodifferentiation-promoting effect on i-PDLSCs. However, on days 14 and 21, different concentrations of i-EXO-GA yielded a higher osteodifferentiation promoting effect than the h-EXO and i-EXO groups. During the comparison of different concentrations of i-EXO-GA, the osteodifferentiation function of low concentration i-EXO-GA was worse than medium and high concentration groups at some time points. The difference between the medium and the high concentration groups was not significant, except for COL-I on day 14. Overall, our study revealed that 200 μg/mL i-EXO-GA could promote osteodifferentiation.

## 4. Discussion

One major tissue engineering application of PDLSCs is alveolar bone regeneration induction [15]. Current evidence suggests that PDLSCs can self-renew and have multi-directional differentiation potential, which are basic characteristics of mesenchymal stem cells [16]. Thus, osteogenic-induced PDLSCs are widely regarded as seed cells for alveolar bone tissue engineering. Although the osteodifferentiation function of PDLSCs is well-established [5], the repair of bone defects during clinical application involves a more complex pathological environment. Periodontitis is widely acknowledged as an important underlying disease that leads to alveolar bone defect and insufficient bone mass [17]. Periodontitis can negatively impact the primary culture of autologous PDLSCs and induce a decline in osteodifferentiation [18]. A study compared PDLSCs derived from normal periodontal tissue and inflammatory periodontal tissues and found that i-PDLSCs showed a weaker osteodifferentiation but better proliferation ability [19]. In our study, the MTT assay found that the proliferation activity of i-PDLSCs was significantly lower than h-PDLSCs. Accordingly, severe periodontitis tissues were selected for primary culture in our study. i-PDLSCs may be subject to damage induced by severe inflammation, leading to a low proliferation activity. Overwhelming evidence substantiates that during periodontitis, PDLSCs can stimulate macrophages to secrete inflammatory factor IL-1β by activating its endoplasmic reticulum stress, thereby mediating bone resorption and inhibiting bone tissue regeneration [20]. We previously established that exosomes of inflammatory PDLSCs were rich in RANKL and TNF-α, which could promote the osteodifferentiation of macrophages, possibly via exosome-mediated intercellular communication prompted pathological bone resorption in periodontitis [21]. In this study, we found that i-PDLSCs had higher expression levels of TNF-α and IL-1β by mRNA sequencing, consistent with cytokine expression in the above studies.

During the MTT assay, when 10 mM GA was used for induction of PDLSCs, similar findings were obtained to previous studies that 1 mM has no cytotoxicity on Vero cells, 4.17 mM has no cytotoxicity on lymphocytes, and 31.76 mM is the half lethal dose of HeLa cells [22,23]. This study demonstrated cytotoxicity on i-PDLSCs cells with GA concentrations above 11.057 mM. It has been established that GA has anti-inflammatory, antioxidant, anti-tumor, anti-platelet aggregation, and bacteriostatic effects [24]. In oral diseases, GA plays an antibacterial role in changing the morphology and structure of dental plaque biofilm, reducing the density of biofilm and killing bacteria in biofilm, and inhibiting the secretion of TNF-α [25]. Thus, it plays an anti-inflammatory role. Therefore, the xipayi mouth rinse, with gallic acid as the main component, is used as a gargle for the clinical treatment of periodontitis [26]. In this study, transcriptome sequencing showed that h-PDLSCs expressed many genes related to osteodifferentiation, and i-PDLSCs cells highly expressed TNF-α and other inflammatory factors, consistent with the function and phenotype reported in previous studies. i-PDLSCs-GA exhibited high expression of genes that promote antioxidant stress, glucose aerobic metabolism, and osteodifferentiation. Previous studies found that GA could inhibit the activation of apoptosis-related proteins, reduce the level of ROS in mitochondria, and downregulate the expression of COX-2 and iNOS [13], thereby reducing the concentration of lipopolysaccharide in vivo [27]. It is well-established that inflammation can cause oxidative stress. This study found that induction culture with GA can improve the oxidative stress of PDSLCs-i-GA and promote aerobic glucose metabolism, proving that GA has a prominent effect on improving the proliferative activity and inflammatory phenotype of PDLSCs.

During analysis of oxidative stress and glycolysis levels in PDSLCs, we found that mitochondria in i-PDLSCs possess a low membrane potential, high oxidative stress, and high glycolysis levels. Current evidence suggests that phagocytosis of neutrophils activated by pathogenic microbial invasion can increase oxygen consumption in periodontitis tissue, and the reperfusion period after hypoxia can lead to increased ROS production in periodontal cells [28]. Hence, following damage induced by the inflammatory response, i-PDLSCs mainly exhibit glycolysis energy consumption and high oxidative stress levels [29]. In contrast, the oxidative stress levels of i-PDLSCs-GA were alleviated after GA induction treatment with downregulated MDA and upregulated SOD levels [30], and the glycometabolism mode gradually changed from glycolysis to aerobic oxidation, comparable to h-PDLSCs [31]. However, little is currently known about glycometabolism during osteodifferentiation. It is widely thought that cells prefer energy produced via glycolysis which is more effective during osteodifferentiation [32,33]. Meanwhile, our findings suggest that i-PDLSCs adopt glycolysis after the mitochondrial membrane potential is decreased and mitochondrial function is impaired by oxidative stress injury. Glycolysis is a prerequisite for the tricarboxylic acid cycle and oxidative phosphorylation. After GA induction, ATP production is increased while the lactate content and activity of glycolytic rate-limiting enzymes are decreased, and i-PDLSCs-GA produce more pyruvate as a substrate for the tricarboxylic acid cycle through glycolysis, instead of lactate as a glycolytic product. Albeit PDLSCs have promising osteodifferentiation potential, the osteodifferentiation potential of i-PDLSCs extracted from periodontitis tissues was not significant. To the best of our knowledge, no study has hitherto investigated the effect of GA on osteodifferentiation of cells. Our transcriptome sequencing results revealed that i-PDLSCs-GA exhibited high expression levels of SOD2 (MnSOD), SIRT3, and other genes, suggesting that the effect of GA on the osteodifferentiation ability of PDLSCs may be mediated through the MnSOD/SIRT3 signaling pathway, which reportedly promotes osteodifferentiation by protecting cells from oxidative stress damage and promoting aerobic metabolism [32].

Exosomes are tiny vesicles containing nucleic acids, proteins, lipids, etc. secreted by cells [34]. Exosomes are well-established to express specific markers, such as CD9, CD63, CD81, ALIX, etc. [35,36]. In the present study, exosome samples with the same protein concentration were used to measure the markers mentioned above but not β-actin, which is consistent with the established characteristics of exosomes. It has been shown that the exosomes of MSCs mediate paracrine/autocrine effects after being taken up by target cells [37], promote the synthesis of growth factors, cytokines, regulatory factors, signal peptides [38], and other bioactive molecules by target cells, and activate the migration of stem cells to the site of tissue damage to participate in repair, as well as regulate the directional differentiation and specific molecular expression of stem cells [39,40]. Current evidence suggests that exosomes contain less immunogenic material than stem cells and enable target and drug delivery adjustment [41]. More importantly, exosomes can be extracted and frozen for storage, allowing easy adjustment of drug delivery concentrations [42,43]. When treating alveolar bone defects associated with periodontitis, stem cells may undergo alterations in cellular functions in the inflammatory microenvironment in vivo, whereas exosomes contain relatively stable components [44]. Thus, exosomes have huge potential for clinical application. Recent studies have also focused on inflammatory bone loss in the alveolar bone associated with periodontitis, using normal PDLSCs-derived exosomes with biomaterials for treatment [45]. This study found that exosomes of normal PDLSCs can inhibit excessive activation of Wnt and promote the osteodifferentiation ability of inflammatory PDLSCs [43]. Using normal PDLSCs exosomes with matrix gelma/β-tricalcium phosphate composites can result in cell-free therapy for bone loss caused by periodontitis [46,47]. In this study, we concluded that although h-EXO has a good osteodifferentiation ability, periodontitis is the most common underlying disease in clinical patients with alveolar bone defects, which makes it difficult to provide h-PDLSCs, while the primary-acquired ones are mostly i-PDLSCs with an inflammatory phenotype. For the primary culture of inflammatory tissue cells, different commercial media containing growth factors such as FGF are available but expensive, while in vitro induction culture of GA is relatively cheaper, yielding i-EXO-GA with good osteodifferentiation ability, suggesting it has huge prospects for application in the treatment of alveolar bone defects associated with periodontitis.

Indeed, further exploration of the expression of miRNA and protein components in i-EXO-GA is warranted, given that the mechanism of its contribution to osteodifferentiation remains unclear. Moreover, the effectiveness of i-EXO-GA treatment of alveolar bone defects associated with periodontitis with biomaterials warrants further investigation in animal studies.

## 5. Conclusions

In this study, we substantiated that GA could improve the proliferative activity and oxidative stress level of i-PDLSCs, increase aerobic metabolism in cells, and improve the function of its exosome to contribute to osteodifferentiation. This study provides a theoretical basis and technical method for the primary culture of PDLSCs and induction culture before exosome acquisition. Accordingly, i-EXO-GA has huge prospects for application in treating alveolar bone defects associated with periodontitis.

## Figures and Tables

**Table 1:** Gene primer sequences for RT-PCR.

Gene	Direction	Primer Sequences
NDUFB-3	Forward	TCAGATTGCTGTCAGACATGG
	Reverse	TGGTGTCCCTTCTATCTTCCA
SDHB	Forward	AAATGTGGCCCCATGGTATTG
	Reverse	AGAGCCACAGATGCCTTCTCT
β-tubulin	Forward	CCCAACAATGTGAAGACGG
	Reverse	GCCTCGGTGAACTCCATCT
CS	Forward	CGGCTACCACATCCAAGGAA
	Reverse	GCTGGAATTACCGCGGCT
IDH	Forward	TCACCAAATGGCACCATACGA
	Reverse	GCCAACATGACTTACTTGATCCC

**Table 2:** Gene primer sequences for RT-PCR.

Gene	Direction	Primer Sequences
OCN	Forward	GACCCTCTCTCTGCTCACT
	Reverse	CACCTTACTGCCCTCCTGC
RUNX2	Forward	GTAGAGAGCAGGGAAGAC
	Reverse	GCTTGGATTAGGGAGTCAC
CoL-I	Forward	TTCTCCTGGCAAAGACGGAC
	Reverse	TTGCTGTTGAAGTCGCAGGAG

## Data Availability

The data used to support the findings of this study are included in the manuscript.

## Supplementary Materials

The following supporting information can be downloaded at: https://www.mdpi.com/article/10.3390/life12091392/s1, Table S1: Lane information of western blot.
