# Supplementary material for: Gallic Acid Ameliorates the Inflammatory State of Periodontal Ligament Stem Cells and Promotes Pro-Osteodifferentiation Capabilities of Inflammatory Stem Cell-Derived Exosomes
Source: Life (Basel). 2022 Sep 6;12(9):1392. doi: 10.3390/life12091392 (PMC9501550; doi:10.3390/life12091392)
Supplement: Supplementary file 1 [file life-12-01392-s001.zip › life-1856776-supplementary.pdf]

Table S1：Lane information of western blot

Lane	Protein concentration of samples
1: h-PDLSCs	1 mg/ml
2: i-PDLSCs	1 mg/ml
3: i-PDLSCs-GA	1 mg/ml
4: h-EXO	1 mg/ml
5: i-EXO	1 mg/ml
6: i-EXO-GA	1 mg/ml
7: i-EXO-GA (low concentration)	0.3-0.5 mg/ml

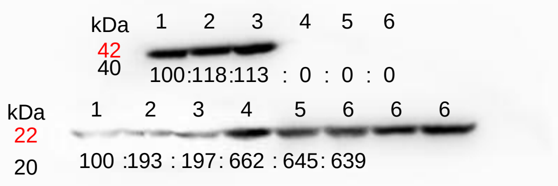

The original image of fig. 4B (β-actin and CD9)

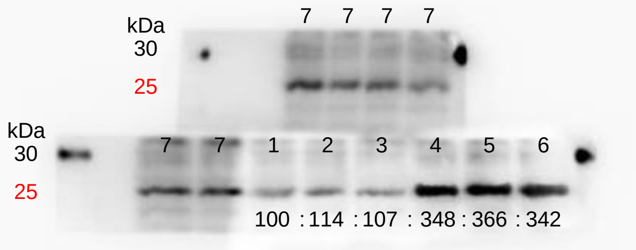

The original image of fig. 4B (CD81)

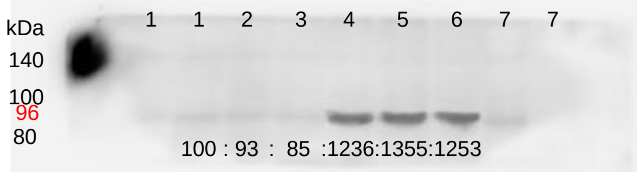

The original image of fig. 4B (ALIX)

The original image of fig. 4B
